# Clinical significance of respiratory bacteria and mycobacteria isolates in adult bronchiectasis in Taiwan

**DOI:** 10.1183/23120541.00865-2024

**Published:** 2025-07-14

**Authors:** Chia-Ling Chang, Chau-Chyun Sheu, Ping-Huai Wang, Meng-Heng Hsieh, Wu-Huei Hsu, Ming-Tsung Chen, Wei-Fan Ou, Yu-Feng Wei, Tsung-Ming Yang, Chou-Chin Lan, Cheng-Yi Wang, Chih-Bin Lin, Ming-Shian Lin, Yao-Tung Wang, Ching-Hsiung Lin, Shih-Feng Liu, Meng-Hsuan Cheng, Yen-Fu Chen, Wen-Chien Cheng, Chung-Kan Peng, Ming-Cheng Chan, Ching-Yi Chen, Lun-Yu Jao, Ya-Hui Wang, Chi-Jui Chen, Shih-Pin Chen, Yi-Hsuan Tsai, Shih-Lung Cheng, Horng-Chyuan Lin, Jung-Yien Chien, Hao-Chien Wang

**Affiliations:** 1 Department of Internal Medicine, National Taiwan University Hospital Hsin-Chu Branch, Hsin-Chu, Taiwan; 2 Graduate Institute of Clinical Medicine, College of Medicine, National Taiwan University, Taipei, Taiwan; 3 Division of Pulmonary and Critical Care Medicine, Department of Internal Medicine, Kaohsiung Medical University Hospital, Kaohsiung, Taiwan; 4 Department of Internal Medicine, School of Medicine, College of Medicine, Kaohsiung Medical University, Kaohsiung, Taiwan; 5 Division of Thoracic Medicine, Far Eastern Memorial Hospital, New Taipei City, Taiwan; 6 Department of Thoracic Medicine, Chang Gung Memorial Hospital at Linkou, Taoyuan, Taiwan; 7 College of Medicine, Chang Gung University, Taoyuan, Taiwan; 8 Division of Pulmonary and Critical Care Medicine, Department of Internal Medicine, China Medical University Hospital, Taichung, Taiwan; 9 Critical Medical Center, China Medical University Hospital, Taichung, Taiwan; 10 School of Medicine, China Medical University, Taichung, Taiwan; 11 Division of Pulmonary and Critical Care Medicine, Department of Internal Medicine, Tri-Service General Hospital, National Defense Medical Center, Taipei, Taiwan; 12 Division of Chest Medicine, Department of Internal Medicine, Taichung Veterans General Hospital, Taichung, Taiwan; 13 Division of Chest Medicine, Department of Internal Medicine, E-Da Cancer Hospital, I-Shou University, Kaohsiung, Taiwan; 14 School of Medicine for International Students, College of Medicine, I-Shou University, Kaohsiung, Taiwan; 15 Division of Pulmonary and Critical Care Medicine, Chiayi Chang Gung Memorial Hospital, Chiayi, Taiwan; 16 Division of Pulmonary Medicine, Department of Internal Medicine, Taipei Tzu Chi Hospital, Buddhist Tzu Chi Medical Foundation, New Taipei City, Taiwan; 17 Department of Internal Medicine, Cardinal Tien Hospital and School of Medicine, College of Medicine, Fu Jen Catholic University, New Taipei City, Taiwan; 18 Division of Pulmonary Medicine, Department of Internal Medicine, Hualien Tzu Chi Hospital, Buddhist Tzu Chi Medical Foundation, Hualien, Taiwan; 19 School of Medicine, Tzu Chi University, Hualien, Taiwan; 20 Division of Pulmonary Medicine, Department of Internal Medicine, Chia-Yi Christian Hospital, Chia-Yi, Taiwan; 21 Division of Pulmonary Medicine, Department of Internal Medicine, Chung Shan Medical University Hospital, Taichung, Taiwan; 22 School of Medicine, Chung Shan Medical University, Taichung, Taiwan; 23 Department of Internal Medicine, Division of Chest Medicine, Changhua Christian Hospital, Changhua, Taiwan; 24 Institute of Genomics and Bioinformatics, National Chung Hsing University, Taichung, Taiwan; 25 PhD Program in Translational Medicine, National Chung Hsing University, Taichung, Taiwan; 26 Division of Pulmonary and Critical Care Medicine, Department of Internal Medicine, Kaohsiung Chang Gung Memorial Hospital, Kaohsiung City, Taiwan; 27 Department of Respiratory Therapy, Kaohsiung Chang Gung Memorial Hospital, Kaohsiung City, Taiwan; 28 Department of Respiratory Therapy, College of Medicine, Kaohsiung Medical University, Kaohsiung, Taiwan; 29 Department of Internal Medicine, National Taiwan University Hospital, Yunlin Branch, Douliu, Taiwan; 30 Thoracic Medicine Center, Department of Medicine and Surgery, National Taiwan University Hospital Yunlin Branch, Yunlin County, Taiwan; 31 School of Post Baccalaureate Medicine, College of Medicine, National Chung Hsing University, Taichung, Taiwan; 32 Medical Research Center, Cardinal Tien Hospital and School of Medicine, College of Medicine, Fu Jen Catholic University, New Taipei City, Taiwan; 33 Department of Pulmonary Medicine, Lee's Clinic, Pingtung, Taiwan; 34 Department of Chemical Engineering and Materials Science, Yuan Ze University, Taoyuan City, Taiwan; 35 Department of Respiratory Therapy, Chang Gung Memorial Hospital at Linkou, Taoyuan, Taiwan; 36 Department of Internal Medicine, National Taiwan University Hospital, National Taiwan University College of Medicine, Taipei, Taiwan; 37 Department of Medicine National Taiwan University Cancer Center, National Taiwan University College of Medicine, Taipei, Taiwan; 38 J-Y. Chien and H-C. Wang contributed equally to this article as lead authors and supervised the work

## Abstract

**Background:**

The clinical impact of bacterial and mycobacterial isolates on bronchiectasis remains uncertain.

**Methods:**

Patients with bronchiectasis at 16 hospitals in Taiwan were recruited with a 1-year follow-up. The patients were classified into six groups: Group 1, *Pseudomonas aeruginosa*; Group 2, *Klebsiella pneumoniae*; Group 3, other bacteria; Group 4, non-tuberculous mycobacteria (NTM); Group 5, daily sputum without bacterial or NTM colonisation; and Group 6, dry bronchiectasis.

**Results:**

In total, 1416 patients (mean age 67 years; 43% males) were included. The mean modified Reiff score was 5 (range 1–18). 59% (829 patients) had sputum, whereas the remaining did not. The proportions of bacteria and NTM cultured from sputum within 1 year of observation were 27% (381/1416) and 15% (202/1416), respectively. The most common bacterial isolate was *P. aeruginosa* (13%), followed by *K. pneumoniae* (7%). 26% of the patients experienced severe exacerbations at least once within the year. The 1-year all-cause mortality rate was 3%. Patients with sputum exhibited a higher rate of severe exacerbations compared to patients with dry bronchiectasis, regardless of the presence of bacteria or NTM (p<0.001). Patients with bacterial colonisation had a higher mortality rate (p<0.001). Further, the highest mortality rate was observed among those with *K. pneumoniae* colonisation (hazard ratio (HR) 8.39 (95% CI 2.39–29.49)), followed by individuals colonised with other bacteria (HR 8.04 (95% CI 2.36–27.38)) and *P. aeruginosa* (HR 7.83 (95% CI 2.45–25.03)). Additionally, old age was an independent risk factor (HR 2.72 (95% CI 1.19–6.18)).

**Conclusion:**

*K. pneumoniae* was more frequently isolated from patients with bronchiectasis in Taiwan compared to Western countries and was associated with unfavourable clinical outcomes.

## Shareable abstract

**In Taiwan, not only *Pseudomonas aeruginosa* but also *Klebsiella pneumoniae* were frequently isolated from patients with bronchiectasis and the highest mortality rate was observed among those with *K. pneumoniae*** https://bit.ly/3DODC9k

## Introduction

Bronchiectasis is a chronic lung disease characterised by radiologically abnormal bronchial wall dilation and is considered heterogeneous with various aetiologies [1]. It is a global health problem with significant healthcare expenses due to exacerbations and hospitalisations [2]. Notably, the characteristics of bronchiectasis differ geographically [3–8] and can occur in all age groups [9]. The patients show different clinical presentations, comorbidities, lung function patterns and microbiological findings. This high degree of heterogeneity makes treatment more challenging [10]. One of the therapeutic strategies for bronchiectasis focuses on controlling chronic bacterial infections using antibiotics [11, 12]. Studies in Europe have noted the importance of *Pseudomonas aeruginosa* and *Haemophilus influenzae* [4, 7, 13, 14]. However, some studies in Asia have found a relatively high prevalence of *Klebsiella pneumoniae* [15, 16], although its clinical impact remains uncertain. Furthermore, the prevalence and clinical impact of mycobacteria in the Asia–Pacific region remain unclear. Therefore, this study aimed to investigate the roles of bacteria and mycobacteria in bronchiectasis in Taiwan.

## Materials and methods

### Study design and participants

The study was a multicentre retrospective observational cohort study. Patients diagnosed with bronchiectasis between January 2017 and June 2020, who attended at least two follow-up visits at the chest clinics of 16 hospitals in Taiwan, were enrolled based on the 2017 European Respiratory Society guidelines [1]. These included patients who underwent sputum cultures for both bacteria and mycobacteria as well as those without sputum. Patients <20 years of age were excluded. All enrolled patients were followed up for 1 year. This study was approved by the Institutional Review Board of National Taiwan University Hospital (202110079RINC).

### Microbiological examination

Sputum samples were cultured according to standard protocols to identify common respiratory pathogens. Culturing involved inoculation of blood/eosin methylene blue agar and chocolate agar, followed by incubation in a 5% CO_2_-enriched atmosphere at 35°C. Bacterial isolates were identified using standard biochemical techniques, along with mass spectrometry analysis on a Bruker Biotyper matrix-assisted laser desorption/ionisation time-of-flight mass spectrometry system (Bruker, Billerica, MA, USA). Smear microscopy and *Mycobacterium* cultures were performed as previously described [17]. Smears were examined using Ziehl–Neelsen staining. All isolates were cultured on Löwenstein–Jensen medium slants (Becton Dickinson, Franklin Lakes, NJ, USA).

Among 2753 patients, 372 with only mycobacterial cultures, 111 with only bacterial cultures and 854 patients who could produce sputum but did not undergo microbiological examinations were excluded, resulting in a final total of 1416 patients (supplementary figure E1).

### Patient grouping

Patients were grouped according to the microbiological isolates in their sputum, and satisfaction with the definition of chronic infection [18] was not necessary. Similar to most previous studies from several countries [3, 5, 6, 13], *P. aeruginosa* was the most common bacterial isolate and *K. pneumoniae* ranked as the second most common bacteria isolated in Taiwan. We further divided patients into six groups for analysis: Group 1, *P. aeruginosa*; Group 2, *K. pneumoniae*; Group 3, other bacteria; Group 4, NTM; Group 5, daily sputum without bacterial or NTM colonisation; and Group 6, dry bronchiectasis.

Owing to the higher pathogenicity of bacteria compared to that of NTM, patients with bronchiectasis were classified into six groups according to the following criteria. If *P. aeruginosa* was detected in the sputum, the patient was allocated to Group 1, regardless of other concurrent bacterial growth. Patients with sputum containing only *K. pneumoniae* or both *K. pneumoniae* and other bacteria, excluding *P. aeruginosa*, were classified into Group 2. Those with sputum samples containing bacteria other than *P. aeruginosa* and *K. pneumoniae* were allocated to Group 3. Patients with sputum containing NTM without bacterial isolates were classified into Group 4, regardless of species identification and compatibility with the NTM pulmonary disease diagnostic criteria [19]. Patients with microbe-free sputum were assigned to Group 5. Patients without sputum were assigned to Group 6.

### Assessments

After enrolment, we obtained the following information through a comprehensive retrospective medical review: demographics, clinical manifestations, comorbidities, laboratory testing, lung function, microbiology and radiology. The 1-year follow-up included details regarding the frequency of severe exacerbations and survival status after enrolment.

### Definitions

High-resolution computed tomography scans of the chest were reviewed by a trained chest specialist and thoracic radiologist; both were blinded to the clinical data. The extent and severity of bronchiectasis was classified using a modified Reiff score [20]. This scoring system (maximum score 18) assesses the number of lobes involved and the degree of dilatation. Higher scores indicate greater disease involvement.

Lung function testing was performed according to the technical standards described by the European Respiratory Society/American Thoracic Society [21], and percentage predicted forced expiratory volume in 1 s (FEV_1_) was calculated based on reference values for Taiwanese patients [22]. Airflow obstruction was defined as an FEV_1_/forced vital capacity ratio <0.7 [23]. Sputum was collected from the patients through spontaneous sputum expectoration or bronchial lavage, as determined by the clinician. The timing of bronchoscopy and bronchoalveolar lavage was determined by clinical physicians.

An exacerbation was diagnosed when at least three signs among worsened cough and sputum, increased sputum purulence, breathlessness, exercise intolerance, fatigue, malaise, haemoptysis, and a change in bronchiectasis treatment deemed necessary by the clinician were present for ≥48 h [24]. Severe exacerbation was defined as hospitalisation because of an acute exacerbation.

### Statistical analysis

Statistical analyses were performed using SPSS Statistics version 25.0 (IBM, Armonk, NY, USA). Continuous variables were reported as means with standard deviations. Between-group differences in continuous variables were compared using one-way ANOVA. Categorical variables were expressed as percentages (numbers/total numbers). Differences between categorical variables were compared using the Chi-squared test. Bonferroni correction was used for multiple comparisons. Mortality was assessed using Kaplan–Meier analysis and compared using the log-rank test. Prognostic factors for mortality were estimated using Cox proportional hazards analysis. Variables with p<0.10 in the univariate analyses were included in the multivariate analysis. All statistical tests were two-sided. Statistical significance was set at p<0.05.

## Results

In total, 1416 patients with bronchiectasis were enrolled in this study. The mean patient age was 67 years (range 20–98 years). Men comprised 43% (603/1416) of all patients and 27% (352/1278) were smokers. The most common comorbidity related to lung conditions was a history of pneumonia (39%), followed by COPD (32%). The mean modified Reiff score was 5 (range 1–18). An eosinophil count >300 cells·μL^−1^ was seen in 12% (134/1085). Of the 762 patients who underwent pulmonary function tests, 249 (33%) had airflow obstruction. Within 1 year, 26% of the patients experienced severe exacerbations. The 1-year all-cause mortality rate was 3%. The clinical characteristics of the patients are summarised in table 1.

**TABLE 1:** Characteristics of patients with bronchiectasis (n=1416)

**Demographics**	
Age (years)	67±12
≥65 years	56.8 (804/1416)
Men	42.6 (603/1416)
BMI (kg·m^−2^) (n=1361)	22±4
Smoker	27.4 (352/1287)
**Comorbidity**	
History of pneumonia	39.3 (556/1416)
COPD	32.2 (456/1416)
Asthma	17.5 (248/1416)
Old pulmonary tuberculosis	16.0 (226/1416)
ABPA	0.6 (9/1416)
Hypertension	33.1 (468/1416)
Type 2 diabetes mellitus	16.4 (232/1416)
GORD (diagnosed through gastroscopy)	19.6 (278/1416)
Rheumatoid arthritis	2.5 (35/1416)
Sjögren's syndrome	2.3 (33/1416)
Systemic lupus erythematosus	0.6 (9/1416)
Inflammatory bowel disease	0.2 (3/1416)
AIDS	0.1 (1/1416)
Primary ciliary dyskinesia	0 (0/1416)
**Radiological status**	
Modified Reiff score	5±3
**Laboratory data**	
Eosinophil count ≥300 cells·μL^−1^	12.4 (134/1085)
**Functional status**	
Obstruction (FEV_1_/FVC <0.7)	32.7 (249/762)
**Severe exacerbation**	
≥1 times	26.0 (368/1416)
**Death**	3.3 (43/1317)

Data are presented as mean±sd or % (n/N). BMI: body mass index; ABPA: allergic bronchopulmonary aspergillosis; GORD: gastro-oesophageal reflux disease; FEV_1_: forced expiratory volume in 1 s; FVC: forced vital capacity.

**FIGURE 1:**
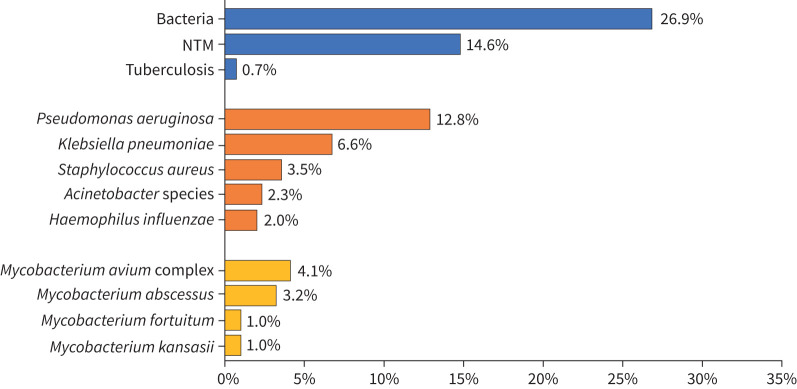
Microbiology in bronchiectasis. NTM: non-tuberculous mycobacteria.

In total, 381 (27%) patients had at least one type of bacterium in their sputum. The most frequently isolated organism was *P. aeruginosa* (13%), followed by *K. pneumoniae* (7%) and *Staphylococcus aureus* (4%). A total of 10 (1%) patients had sputum cultures positive for *Mycobacterium tuberculosis* (tuberculosis), while 202 (15%) had sputum cultures positive for NTM, of which 37% (74/202) had positive sputum smears. Notably, *Mycobacterium avium* complex (MAC) (4%) was the most common, followed by *Mycobacterium abscessus* (3%). Supplementary table E1 and figure 1 show the sputum culture results.

**FIGURE 2:**
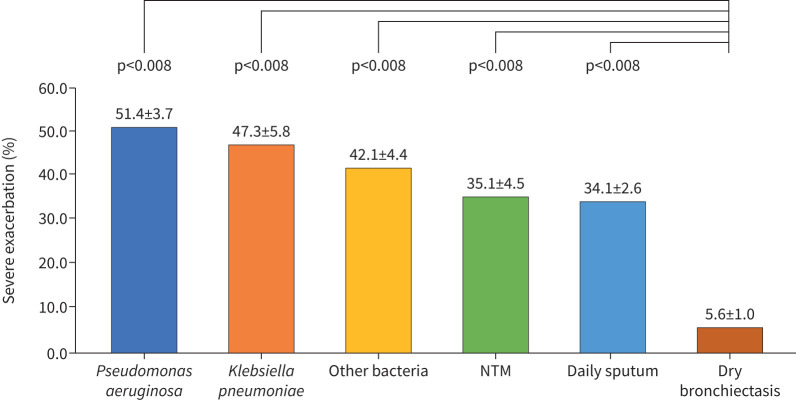
Severe exacerbation of patients with bronchiectasis. Bonferroni-adjusted p-value <0.008. NTM: non-tuberculous mycobacteria.

**FIGURE 3:**
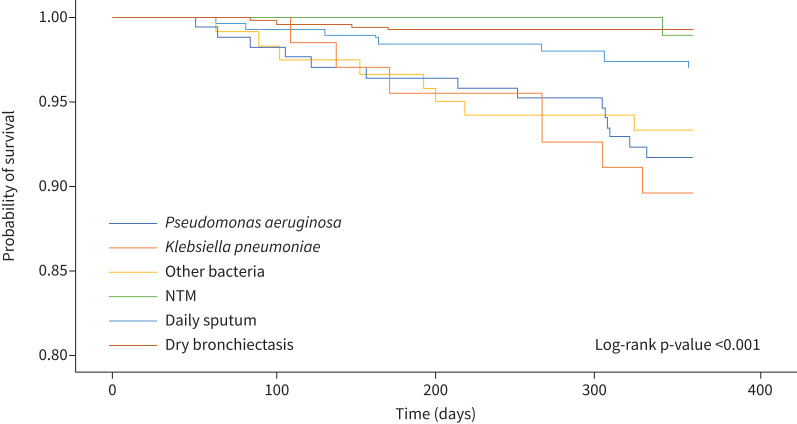
Survival analysis of patients with bronchiectasis. NTM: non-tuberculous mycobacteria.

Compared to patients with dry bronchiectasis (Group 6), patients with sputum exhibited a higher rate of severe exacerbations (Groups 1–5), regardless of the presence of bacteria and/or NTM (p<0.001) (table 2 and figure 2). Patients with bacterial colonisation (Groups 1–3) had a higher 1-year mortality rate than patients with dry bronchiectasis (Group 6) (p<0.001) (table 2). Kaplan–Meier analysis showed a significant difference in 1-year mortality among the six groups (log-rank test p<0.001) (figure 3). Table 3 shows the results of our univariate and multivariate Cox proportional hazards analyses for 1-year mortality. Older age (hazard ratio (HR) 2.72 (95% CI 1.19–6.18); p=0.017) was an independent factor for 1-year mortality. Compared to patients with dry bronchiectasis (Group 6), those with bacterial colonisation (Groups 1–3) had a higher mortality risk. In particular, patients with *K. pneumoniae* colonisation (Group 2) (HR 8.39 (95% CI 2.39–29.49)) demonstrated a higher 1-year mortality rate than patients with *P. aeruginosa* colonisation (Group 1) (HR 7.83 (95% CI 2.45–25.03)) or other bacterial colonisation (Group 3) (HR 8.04 (95% CI 2.36–27.38)).

**TABLE 2:** Clinical characteristics of the different patient groups

	Group 1 *Pseudomonas aeruginosa* (n=181)	Group 2 *Klebsiella pneumoniae* (n=74)	Group 3 Other bacteria (n=126)	Group 4 NTM (n=111)	Group 5 Daily sputum without bacterial or NTM colonisation (n=337)	Group 6 Dry bronchiectasis (n=587)	p-value
**Demographics**							
Age ≥65 years	65.7 (119/181)^#^	70.3 (52/74)^#^	54.8 (69/126)	64.0 (71/111)*	55.5 (187/337)	52.1 (306/587)	0.002
Men	40.3 (73/181)	64.9 (48/74)^#^	42.1 (53/126)	30.6 (34/111)*	40.4 (136/337)	44.1 (259/587)	<0.001
Smoker	24.2 (39/161)	38.9 (28/72)	23.9 (27/113)	19.8 (20/101)	26.0 (81/311)	29.7 (157/529)	0.053
**Comorbidity**							
History of pneumonia	63.5 (115/181)^#^	54.1 (40/74)^#^	53.2 (67/126)^#^	55.0 (61/111)^#^	39.8 (134/337)^#^	23.7 (139/529)	<0.001
COPD	44.2 (80/181)^#^	48.6 (36/74)^#^	35.7 (45/126)*	20.7 (23/111)	35.0 (118/337)*	26.2 (154/587)	<0.001
Asthma	21.0 (38/181)	16.2 (12/74)	15.9 (20/126)	12.6 (14/111)	21.7 (73/337)	15.5 (91/587)	0.096
Old pulmonary tuberculosis	24.3 (44/181)^#^	17.6 (13/74)	15.1 (19/126)	15.3 (17/111)	18.1 (61/337)*	12.3 (72/587)	0.005
**Radiological status**							
Modified Reiff score	7±4^#^	5±3	5±4*	6±4^#^	5±3*	4±3	<0.001
**Functional status**							
Obstructive pattern	50.5 (54/107)^#^	42.2 (19/45)*	31.1 (19/61)	23.4 (15/64)	35.2 (69/196)*	25.3 (73/289)	<0.001
**Laboratory data**							
Eosinophil count ≥300 cells·μL^−1^	9.8 (16/163)	19.0 (12/63)	15.9 (17/107)	9.5 (9/95)	13.9 (36/286)	11.1 (41/371)	0.256
**Severe exacerbation**							
≥1 times	51.4 (93/181)^#^	47.3 (35/74)^#^	42.1 (53/126)^#^	35.1 (39/111)^#^	34.1 (115/337)^#^	5.6 (33/587)	<0.001
**1-year all-cause mortality**	8.3 (14/168)^#^	10.4 (7/67)^#^	6.7 (8/120)^#^	1.0 (1/108)	2.9 (9/309)*	0.7 (4/553)	<0.001

Data are presented as mean±sd or % (n/N). NTM: non-tuberculosis mycobacteria. ^#^: Bonferroni-adjusted p-value <0.008 (reference: dry bronchiectasis). *: p<0.05 (reference: dry bronchiectasis).

**TABLE 3:** Risk factors associated with 1-year all-cause mortality of patients with bronchiectasis

	Mean survival time (95% CI) (days)	Univariate		Multivariate	
		Hazard ratio (95% CI)	p-value	Hazard ratio (95% CI)	p-value
**Demographics**					
Age					
<65 years	362.84 (361.05–364.63)	1		1	
≥65 years	357.15 (354.19–360.11)	3.42 (1.59–7.37)	0.002	2.72 (1.19–6.18)	0.017
Sex					
Female	361.83 (359.93–363.72)	1		1	
Men	356.67 (353.21–360.12)	2.81 (1.48–5.32)	0.002	1.39 (0.60–3.21)	0.438
Smoking					
Non-smoker	361.10 (359.21–362.99)	1		1	
Smoker	355.15 (350.10–360.19)	2.61 (1.41–4.81)	0.002	1.66 (0.77–3.60)	0.198
**Comorbidity**					
History of pneumonia					
No	361.32 (359.39–363.52)	1		1	
Yes	356.96 (353.35–360.57)	2.42 (1.31–4.46)	0.005	1.55 (0.80–3.00)	0.196
COPD					
No	362.11 (360.45–363.76)	1		1	
Yes	354.35 (349.84–358.85)	4.05 (2.16–7.59)	<0.001	1.90 (0.94–3.82)	0.072
Asthma					
No	358.81 (356.61–361.01)	1			
Yes	363.46 (361.93–364.98)	0.62 (0.24–1.57)	0.310		
Old pulmonary tuberculosis					
No	359.14 (357.01–361.28)	1			
Yes	362.16 (359.72–364.61)	1.22 (0.56–2.62)	0.620		
**Radiological status**					
Modified Reiff score		1.00 (0.92–1.09)	0.979		
**Groups**					
Dry bronchiectasis	363.28 (361.58–364.98)	1		1	
*Pseudomonas aeruginosa*	352.13 (344.26–360.01)	11.93 (3.93–36.24)	<0.001	7.83 (2.45–25.03)	0.001
*Klebsiella pneumoniae*	350.70 (338.94–362.47)	15.01 (4.39–51.27)	<0.001	8.39 (2.39–29.49)	0.001
Other bacteria	351.97 (342.51–361.43)	9.53 (2.87–31.66)	<0.001	8.04 (2.36–27.38)	0.001
NTM	364.78 (364.36–365.21)	1.35 (0.15–12.07)	0.789	1.18 (0.13–10.74)	0.822
Daily sputum	360.34 (356.77–363.91)	4.09 (1.26–13.28)	0.019	3.17 (0.95–10.60)	0.061

NTM: non-tuberculosis mycobacteria.

## Discussion

This study analysed data from a large-scale registry of Taiwanese patients with bronchiectasis. The most common comorbidity of lung conditions in this study was a history of pneumonia (39%). The proportions of bacteria, *M. tuberculosis* and NTM cultured from sputum samples within 1 year were 27%, 1% and 15%, respectively. The most common bacterial isolate was *P. aeruginosa*, followed by *K. pneumoniae*, whereas the most common NTM isolate was MAC. The incidence rates of severe exacerbation and mortality within 1 year were 26% and 3%, respectively. Patients with sputum exhibited a higher rate of severe exacerbations compared to patients with dry bronchiectasis, regardless of the presence of bacteria or NTM. Moreover, patients with bacterial colonisation showed a higher 1-year mortality rate than patients without sputum. Notably, patients with *K. pneumoniae* colonisation had the highest 1-year mortality rate.

*P. aeruginosa* is one of the most common pathogens in almost all countries [3, 5, 6, 13]. The presence of *P. aeruginosa* in sputum is associated with acute exacerbations and mortality [4, 14]. *H. influenzae* is also commonly isolated in Europe [7, 13]. However, *K. pneumoniae* has been classified under the Enterobacteriaceae family in these studies and its incidence has not been calculated separately [7, 13]. This suggests that *K. pneumoniae* is not sufficiently prevalent to warrant further attention. However, *K. pneumoniae* was the second most common isolate in our study, consistent with findings from Asian countries (22.4% in South Korea, 14% in Thailand and 1.3% in northern China, all of which rank second only to *P. aeruginosa*) [15, 16, 25], and was higher than in the USA [5]. Furthermore, a bronchiectasis registry study from India revealed that patients infected with *K. pneumoniae* (62%) experienced a 3.13-fold increase in mortality after adjusting for age, sex, FEV_1_, smoking status and exacerbations [4]. Indian patients with *K. pneumoniae* infections tended to be younger, were more frequently male and had more severe bronchiectasis. They were also more likely to have underlying COPD, be current smokers, and report worse symptoms and quality of life. Additionally, two-thirds of these patients had a history of hospitalisation in the previous year and one-third had associated diabetes. These findings suggest that *K. pneumoniae* is a significant pathogen associated with severe disease and poor outcomes in India [4]. Our study further demonstrated that the mortality rates of patients with *K. pneumoniae* (Group 2) showed a higher increase (8.39- *versus* 7.83-fold increase) than those in patients with *P. aeruginosa* (Group 1) when compared to patients with dry bronchiectasis (Group 6). Thus, unlike in Western countries, *K. pneumoniae* appears to be a significant bacterium in Asian countries.

Recently, a high prevalence of NTM isolation has been reported in patients with bronchiectasis [6, 26]. Between 2006 and 2021, the global prevalence of NTM colonisation in adults with bronchiectasis was estimated to be ∼10% [27], with higher rates observed in South Korea (25%) [28] and China (23%) [29], and lower rates in Greece (1%) [30] and Pakistan (1%) [31]. A recent study from India reported NTM isolates in only 0.4% of patients with bronchiectasis; however, only about half of the patients (59%) underwent sputum examination and no mycobacteria-specific culture was performed, which may have contributed to the extremely low rate of NTM detection [3]. In our study, patients without mycobacterial cultures were excluded; therefore, our findings may have overestimated the prevalence of NTM pulmonary disease in bronchiectasis. Furthermore, a recently published study from the US Bronchiectasis and NTM Research Registry found that the annual bronchiectasis exacerbation rate was lower in patients with NTM colonisation or infection [32]. However, in our study, patients with NTM without other bacterial isolates (Group 4) experienced more exacerbation than patients with dry bronchiectasis (Group 6). This difference may be attributed to the lower proportion of patients receiving NTM treatment in our study (only 16.2% of Group 4 patients). Further prospective studies are thus required to confirm these findings.

Bronchiectasis is a heterogeneous disease, which makes it difficult to assess the risk of exacerbations using a single indicator. A thorough assessment of bronchiectasis symptoms can serve as a useful indicator to identify patients at increased risk of future exacerbations [33]. Sputum purulence, frequency of exacerbations and severity of daily symptoms can be used as indicators to assess bronchiectasis activity [34, 35]. A European study indicated that sputum colour, which reflects neutrophilic inflammation [36], was associated with severe exacerbations and mortality rates in bronchiectasis. The more purulent the sputum, the higher were the rates of severe exacerbations and mortality [37]. Patients with bronchiectasis who had stronger symptoms had a 1.74-fold increased risk of exacerbations within 1 year compared to patients with weaker symptoms [38].

Our study indicated that patients with sputum (Groups 1–5) exhibited a higher rate of severe exacerbations, regardless of the presence of bacteria or NTM, than patients with dry bronchiectasis (Group 6). Overall, assessing the symptoms in bronchiectasis is very important.

Bronchiectasis is characterised by stable periods interrupted by episodes of significant symptom worsening, referred to as exacerbations [39]. The mechanisms of exacerbations have been investigated by assessing both microbial and non-microbial triggers, along with the associated comorbidities [40, 41]. This includes examining the effects of external factors, such as infections [41, 42], and internal factors, such as inflammatory endotypes [41, 43]. The severity and frequency of exacerbations are crucial indicators of disease management effectiveness, and are associated with higher mortality rates, reduced lung function and a decline in quality of life [24]. Our study focused on the relationship between individual microbial triggers and severe exacerbations, indicating poor disease control, and aimed to identify tailored treatments for each patient with bronchiectasis.

This study had several limitations. First, because of the retrospective study design, we could not evaluate the bronchiectasis severity score, mild and moderate exacerbations or bronchiectasis status upon obtaining sputum cultures. Second, we could not determine the exact aetiology of bronchiectasis in each patient. Third, bacterial resistance was not investigated in this study. Fourth, all sputum NTM-positive specimens did not undergo further species identification. Fifth, we only grouped patients according to the isolates from sputum specimens, therefore our findings cannot be applied to chronic infections (defined as the presence of the same microorganism in sputum cultures on at least two occasions within 1 year, during periods when bronchiectasis is stable, with each culture separated by at least 3 months [18]). Finally, our study excluded 1337 participants without comprehensive microbiological cultures from a total of 2753 participants, which may have introduced a selection bias into the analysis. Overall, further research is required to validate the results of the present study.

### Conclusion

*K. pneumoniae* is frequently isolated from patients with bronchiectasis in Taiwan compared to those in Western countries and is associated with unfavourable clinical outcomes. This result needs to be further confirmed through prospective studies.

## Supplementary material

**Please note:** supplementary material is not edited by the Editorial Office, and is uploaded as it has been supplied by the author. Supplementary material 00865-2024.SUPPLEMENT
