# Supplementary material for: Clinical significance of respiratory bacteria and mycobacteria isolates in adult bronchiectasis in Taiwan
Source: ERJ Open Res. 2025 Jul 14;11(4):00865-2024. doi: 10.1183/23120541.00865-2024 (PMC12257146; doi:10.1183/23120541.00865-2024)

e-Table 1. Microbiology in patient with bronchiectasis

**Isolate**	**Percentage %**
Any bacteria	26.9 (381/1416)
Pseudomonas aeruginosa	12.8 (181/1416)
Klebsiella pneumoniae	6.6 (94/1416)
Staphylococcus aureus	3.5 (49/1416)
Acinetobacter species	2.3 (32/1416)
Haemophilus influenzae	2.0 (28/1416)
Escherichia coli	1.8 (26/1416)
Enterobacter	1.1 (15/1416)
Streptococcus pneumoniae	0.6 (8/1416)
Moraxella catarrhalis	0.1 (2/1416)
Tuberculosis	0.7 (10/1416)
Any NTM	14.6 (202/1416)
Mycobacterium avium complex	4.1 (58/1416)
Mycobacterium abscessus	3.2 (46/1416)
Mycobacteria fortuitum	1.0 (14/1416)
Mycobacterium kansasii	1.0 (14/1416)
Mycobacteria gordonae	1.0 (14/1416)
Mycobacteria xenopi	0.6 (8/1416)

NTM, non-tuberculosis mycobacterium.

e-Figure 1. Study enrollment screening flowchart.

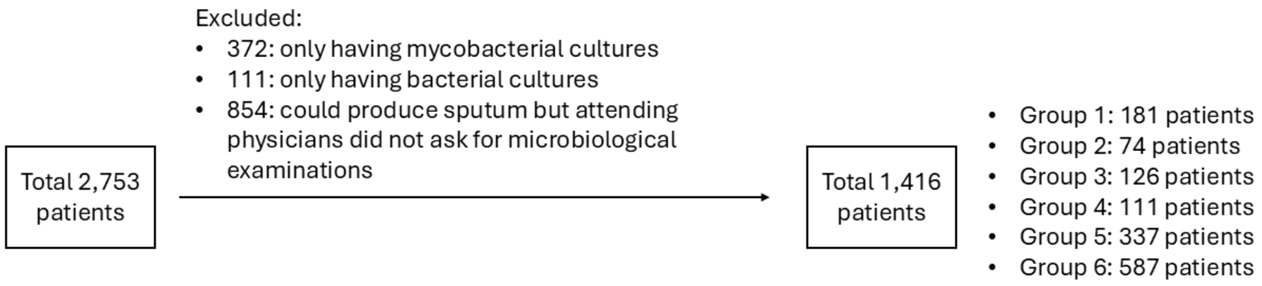

Supplement: Supplementary file 1 [file 00865-2024.SUPPLEMENT.pdf]
